# Dermatologic conditions in internationally adopted children^☆^

**DOI:** 10.1016/j.ijwd.2014.12.003

**Published:** 2015-03-02

**Authors:** Diane L. Whitaker-Worth, Cheryl B. Bayart, Julia Anderson Benedetti

**Affiliations:** a University of Connecticut Health Center, Farmington, Connecticut; b Cleveland Clinic Foundation, Cleveland, Ohio; c Lahey Hospital & Medical Center, Burlington, Massachusetts

**Keywords:** Health care delivery, Skin signs of systemic disease, Infection-bacterial, Infection-fungal, Insect bite/bite reactions, Pediatric dermatology

## Abstract

Over 200,000 children have been adopted into United States (US) families from abroad since the year 2000. Health care providers who care for children adopted internationally should be aware of the spectrum of illnesses seen in this population, and should be prepared to encounter potentially unusual situations. An appreciation for the unique pre-adoption exposures and vulnerabilities inherent in international adoption is critical for proper diagnosis and treatment of this heterogeneous group of children. It is important to consider the impact of potential early childhood stressors such as nutritional, sensory, and emotional deprivation, trauma and abuse, as well as prenatal exposures to drugs, alcohol, and infectious diseases. Providers must also take into account international variation in health care practices, including immunization, treatment, surgical, and hygiene standards. The differential diagnosis for cutaneous eruptions in children adopted internationally is broad and must encompass endemic systemic illnesses with skin manifestations, such as measles, tuberculosis, leprosy, and congenital syphilis, and primary dermatologic diseases such as scabies and bacterial and fungal infections. The importance of maintaining a broad differential and open mind when addressing the dermatologic needs of these children cannot be overemphasized.

## Capsule Summary

• Dermatologists caring for children adopted internationally should be prepared for unusual situations.
• It is important to consider potential iatrogenic and infectious exposures, early physical and psychosocial stressors, and international variation in health care practices.
• The importance of maintaining a broad differential and open mind in these cases cannot be overemphasized.

## Introduction

Over 200,000 children have been adopted into United States (US) families from abroad since the year 2000. International adoptions peaked at nearly 23,000 per year in 2004–2005 and, for a variety of reasons, have steadily declined to approximately 7,000 in 2013 (Intercountry Adoption: Bureau of Consular Affairs, US Department of State). Most children have been adopted from China, Ethiopia, Russia, South Korea, Guatemala and Ukraine (Intercountry Adoption: Bureau of Consular Affairs, US Department of State). There has been a significant increase in the adoption of children with special physical, psychological, or educational needs (Hansen, 2006).

Internationally adopted children are a heterogeneous population, exposed to a broad range of living conditions (Sperling, 2001) and infectious diseases in their birth countries (Staat and Klepser, 2006) (Table 1). Health care systems and documentation vary tremendously by region (Staat and Klepser, 2006). Before leaving their home countries, adoptees undergo basic mandatory screening for specific infectious diseases and serious handicaps that may affect acquisition of a permanent residency visa (Committee on Infectious Diseases, American Academy of Pediatrics, 2009). In addition, the American Academy of Pediatrics recommends that all international adoptees be screened for hepatitis B, syphilis, HIV, tuberculosis, and stool ova and parasites, and receive a complete blood count with red blood cell indices. Additional testing may be warranted depending on potential exposure history and results of initial screening tests (Committee on Infectious Diseases, American Academy of Pediatrics, 2009).

Acutely, children present most often with infection, including intestinal parasites and skin and soft tissue infections (SSTI) (Committee on Infectious Diseases, American Academy of Pediatrics, 2009). Common illnesses may present atypically in international adoptees due to factors such as malnutrition, immune dysregulation, and prior incomplete or ineffective treatment. Inadequately treated conditions may present with unusual complications. Providers should maintain a broad differential diagnosis to include the possibility of endemic, vaccine-preventable, or hospital acquired infections.

## Special Considerations

Early childhood stressors such as sensory and emotional deprivation have been implicated in immune, endocrine and developmental abnormalities. Developmental and growth delays are common among children adopted internationally, especially those who were in foster care or orphanages prior to adoption (Miller and Hendrie, 2000, Miller et al., 2008). Orphanage living, in particular, has been linked to health and developmental problems, as well as nutritional deprivation (Mason and Narad, 2005). The ratio of children to orphanage caregivers can be as high as 60:1, thus, deprivation of basic affection is another concern (Mason and Narad, 2005). Overcrowding in the setting of a high pathogen burden can lead to poor hygiene (Gunnar et al., 2001). Additional stressors include prenatal exposure to drugs, alcohol, and infectious diseases (Mason and Narad, 2005).

## Hypothalamic-pituitary-adrenal (HPA) axis and cortisol regulation

Childhood trauma and deprivation induces measurable alterations in the HPA axis and its stress regulatory hormones. The HPA axis is associated with human growth and cognitive development (Mason and Narad, 2005). In animal models, maternal deprivation can lead to hyper-responsive stress hormones and suppression of growth hormone (GH) (Mason and Narad, 2005). Growth stunting in humans has also been correlated with altered cortisol levels (Mason and Narad, 2005).

Alterations in cortisol regulation have been seen in children with significant stressors, such as living in orphanages with poor standards of care (Carlson and Earls, 1997, Gunnar et al., 2001, Mason et al., 2000). Some post-adoption studies show that suppression of diurnal cortisol variation may be temporary, and early stressors may not affect long-term basal cortisol levels (Kertes et al., 2008). However, one small study of children adopted from a very poor orphanage showed persistently elevated daytime cortisol levels more than 6 years after adoption (Kertes et al., 2008). Aberrations in cortisol correlate with time institutionalized (Kertes et al., 2008). Children adopted younger than 4 months old did not experience the level of cortisol aberrations seen in older institutionalized children (Kertes et al., 2008). These types of hormonal changes can manifest in dermatologic conditions, such as acne and hirsutism, and should be considered in the differential when these present in atypical situations.

## Precocious puberty

Several studies show internationally adopted children to be at up to twenty-fold risk of precocious puberty (Shirtcliff et al., 2009, Teilmann et al., 2006, Teilmann et al., 2009. Dermatologists should consider this when evaluating patients for early onset acne or with potential genetic diseases, such as McCune-Albright syndrome. Precocious puberty risk varies between countries. It is highest for adoptees from India, Africa and South America and negligible for those from Korea (Teilmann et al., 2006). Risk is positively correlated with age at adoption (Teilmann et al., 2006). Sometimes the true age of an adopted child may not be known, due to factors such as poor record keeping or lack of birth information. However, even after adjusting for potential error, a statistically significant increased risk remains (Teilmann et al., 2006).

Potential contributors to precocious puberty in children adopted internationally include pre-adoption dietary factors, hormonal stress response and “catch-up” growth. Decreased growth hormone (GH) secretion and growth stunting may occur as a physiologic response to adverse perinatal conditions or HPA axis dysregulation (Mason and Narad, 2005). When a child’s environment becomes more favorable, GH levels return to normal, and “catch-up” growth occurs (Mason et al., 2000). In adopted children, when this period of “catch up” growth occurs during childhood, as opposed to infancy, the response is no longer physiologic and may incite abnormal endocrine responses, including spikes in insulin-like growth factor, stimulating inappropriate gonadal release of sex hormones (Teilmann et al., 2006).

## Immune Surveillance

Alterations in homeostasis appear to affect immune surveillance, increasing risk of infections such as HSV1 and autoimmune disorders such as alopecia areata, both of which have a higher prevalence in children adopted internationally (Gunnar et al., 2001).

## HSV 1

A 2008 study noted elevated salivary HSV-1 antibody levels without clinical lesions in children with history of institutionalization or abuse, compared with demographically similar controls (Shirtcliff et al., 2009). Elevated antibody levels were thought to result from viral reactivation due to failure of the cellular immune processes that normally maintain the virus in its latent state (Shirtcliff et al., 2009). Levels remained elevated years after adoption, suggesting persistent effect from early life stressors (Shirtcliff et al., 2009).

## Malnutrition

A recent review showed 28% of internationally adopted children to be chronically malnourished and 5% to be acutely malnourished (Mandalakas et al., 2007). This may affect growth, development (Miller et al., 2008, Quarles and Brodie, 1998), and response to vaccinations (Quarles and Brodie, 1998, Staat and Klepser, 2006, Verla-Tebit et al., 2009). The skin may be the presenting sign of nutritional deficits (Gehrig and Dinulos, 2010, Heath and Sidbury, 2006). In cases of zinc, protein, biotin or B vitamin deficiency, prompt nutritional supplementation is essential to prevent sequelae such as growth delay and neurologic, metabolic and hormonal disturbances (Gehrig and Dinulos, 2010, Heath and Sidbury, 2006).

## Immunization status

Immunization status among international adoptees varies widely. Vaccine potencies, dosages, schedules and written records may be incomplete, inaccurate or missing (Staat and Klepser, 2006, Verla-Tebit et al., 2009). Malnutrition can hamper adequate vaccine response. Children may also gain immunity from direct exposure to infectious agents.

BCG (Bacillus Calmette–Guérin), diphtheria, tetanus, pertussis, poliovirus, measles, and hepatitis B vaccines are commonly given throughout much of the world (Committee on Infectious Diseases, American Academy of Pediatrics, 2009). *Haemophilus influenzae*, varicella, *Streptococcus pneumoniae*, hepatitis A, mumps and rubella immunizations are less likely to be administered (Committee on Infectious Diseases, American Academy of Pediatrics, et al., 2009, Quarles and Brodie, 1998). Vaccination status of adoptees from China was significantly improved following the 2005 institution of a national policy providing immunizations to all children free of charge (Van Schaik et al., 2009). A 2005 study of Guatemalan adoptees found that only 28% met American Academy of Pediatrics vaccination standards (Miller et al.). Clinicians should take into account the increased likelihood of a vaccine preventable illness when evaluating this population.

## Miscellaneous scars

When scars are noted in children adopted internationally, lack of information about prior life experiences makes determination of their etiology difficult. Potential causes include trauma, abuse, accidents, and medical interventions in home countries. Safety standards are often poor in orphanages, and even in foster care in many countries. Thus, accidents such as electrical burns may be common. Due to variations in medical procedures, scars due to intravenous line placement or surgery may differ from those seen in the US. The pre-adoption living environment may also render many children susceptible to trauma, abuse and accidental injury.

## Systemic diseases with prominent skin manifestations

### HIV

Due to pre adoption screening, internationally adopted children do not appear to be at increased risk of HIV infection (Chen et al., 2003, Hostetter et al., 1991, Miller, 1999), nor has transmission to a family member been documented (Chen et al., 2003). However, a 1993 study reported an HIV prevalence rate of 20% in one Romanian orphanage, thought to be due to the sharing of medical needles (Hersh et al., 1993, Murray et al., 2005). The reliability of documented HIV screening done in the birth country may vary (Staat, 2002), and screening should be verified. Repeat testing may be considered six months post adoption to rule out exposure immediately prior to adoption or false negative results in younger children (Staat, 2002).

### Measles

Worldwide, measles causes more child deaths than any other vaccine-preventable illness. Children adopted from endemic areas are at increased risk of infection. Family members may be exposed during travel to the child’s birth country or through contact with an infected child following relocation to the US (Broy et al., 2009).

In 2001, ten measles cases were identified among recent adoptees to the US from China and four adoptive family members and close contacts. All cases were linked to a single orphanage (Centers for Disease Control and Prevention, 2002). In 2004, nine Chinese children adopted to the US developed a febrile illness with a measles-like rash. Four cases of measles were confirmed by serology (Wallace et al., 2013). Most recently, in 2006, three American adults were infected during an adoption trip to the Guangdong Province (Centers for Disease Control and Prevention, 2007). Measles continues to pose a problem in the US, with 159 cases reported between January and August 2013. The majority of cases are imported from endemic areas and affect groups of Americans unvaccinated because of philosophical or religious beliefs (Wallace et al., 2013).

Health care providers must consider measles in a patient with febrile illness, erythematous skin eruption, and history of travel to an endemic area or contact with a child adopted internationally (Centers for Disease Control and Prevention, 2004). Active infection can be reliably confirmed by serum ELISA IgM (Broy et al., 2009).

### Leprosy

Although the worldwide burden of leprosy is low, incidence is substantial in many countries. At least one case has been documented in an international adoptee: a 10-year-old girl who had received multidrug treatment in her home country of Ethiopia, but presented with an atypical immune response within a few months of her adoption. The child had completed an unknown course of treatment one month prior to entering the US. Upon adoption, the child had residual hypopigmentation on the right arm and an area of hypesthesia on the ipsilateral thigh. Over the next 3 months, she developed new plaques on the left cheek and ear and right nasal alar swelling. Biopsy results were consistent with an immunologic reaction to residual inert bacteria. All lesions resolved following a two week course of prednisone (King et al., 2009). Risk of transmission to adoptive family members is low, even with active leprosy, as 95% of people are naturally immune and unlikely to develop symptoms (Centers for Disease Control and Prevention, 2004).

### Tuberculosis

An estimated 3–20% of international adoptees will have a positive tuberculin skin test (TST) (Kay and McCarthy, 2009). Risk factors include older age at adoption, BCG vaccination or history of living in an orphanage or similar institution (Mandalakas et al., 2008). Testing is required of all immigrants prior to admission to the United States (Committee on Infectious Diseases: American Academy of Pediatrics, 2009). However, results may be unreliable for adopted children (Mandalakas et al., 2007) due to factors such as malnutrition or stress-induced immunosuppression (Kay and McCarthy, 2009, King et al., 2009). An estimated 20% of international adoptees with negative TSTs upon arrival to the US will test positive within 3 months (Trehan et al., 2008). To our knowledge, no cases of cutaneous tuberculosis have been reported in internationally adopted children (Committee on Infectious Diseases, American Academy of Pediatrics, 2009).

The BCG vaccine is recommended by the WHO for infants in endemic areas, so many children adopted internationally will have received this vaccination prior to adoption. Vaccine-related skin complications have been reported (Bellet and Prose, 2005), and physicians must be aware of these entities (Thakur and Verma, 2011). Normally, a 5-15 mm inflamed, erythematous papule develops 3–4 weeks following BCG vaccination. A central crust forms and then detaches to leave an ulcer, which heals into a scar. Common local reactions include erythema, soreness, ulceration, blistering, and abscess or keloid formation (Trehan et al., 2008, Walker et al., 2009). Atypical and more severe reactions such as lupus vulgaris (Bellet and Prose, 2005, Najem et al., 2009, Thakur and Verma, 2011, Trehan et al., 2008), cutaneous granulomas (Bellet and Prose, 2005, Najem et al., 2009, Thakur and Verma, 2011, Trehan et al., 2008), fixed drug eruption (Thakur and Verma, 2011, Trehan et al., 2008), local hypersensitivity reaction (Thakur and Verma, 2011), and juvenile sarcoidosis (Trehan et al., 2008) are rare, and more likely to occur in children with immunodeficiency (Trehan et al., 2008).

A 2007 case report documented BCG vaccine-induced lupus vulgaris in a 3 year-old girl adopted from China. The girl presented with an inflammatory papule on the preauricular skin, which enlarged into a 2 cm erythematous plaque with focal erosion, purulent yellow exudate and crust, open comedone-like lesions, follicular plugging, scarring, and atrophy (Samuel et al., 2007).

Lupus vulgaris is caused by hematogenous spread of mycobacteria to the skin, however, lupus vulgaris secondary to BCG immunization is rare, occurring at a rate of 5 cases per million vaccinations (Bellet and Prose, 2005, Najem et al., 2009, Thakur and Verma, 2011). Risk is higher with multiple injections, and among females (Thakur and Verma, 2011). Clinically, primary and BCG-induced lupus vulgaris are identical (Najem et al., 2009, Thakur and Verma, 2011). An enlarging inflammatory papule develops into an irregularly shaped, brown-red, scaly plaque with “apple jelly” color displayed on diascopy (Thakur and Verma, 2011). Diameter can range from a few millimeters to over 10 cm (Najem et al., 2009; Thakur and Verma, 2008). Ulceration, atrophy and hypertrophy may be observed. Regional lymphadenopathy is common (Thakur and Verma, 2011).

PCR is the most reliable available means of diagnosing lupus vulgaris, but is only 40% sensitive (Thakur and Verma, 2011). TST is usually positive, but is nonspecific (Najem et al., 2009; Thakur and Verma, 2008). When clinical and histopathological features suggest lupus vulgaris, empiric therapy may be appropriate (Najem et al., 2009).

### Congenital Syphilis

Global incidence of congenital syphilis is estimated at 700,000–1.5 million cases per year (Krüger and Malleyeck, 2010). Resource-poor countries are disproportionately affected (Samuel et al., 2007), however, recent resurgences have been reported in nations such as China and Canada (Zhu et al., 2010). Congenital syphilis should be included in the differential diagnosis of an internationally adopted infant with skin eruptions, particularly if systemic signs of syphilis are present or the mother’s prenatal care history is questionable or unknown (Samuel et al., 2007).

Neonates often present with treponemal sepsis, low birth weight, and hepatosplenomegaly. Skin and mucous membrane lesions may include purulent nasal discharge, jaundice, a morbilliform eruption, bullae, or condyloma lata. Failure to thrive, anemia, syphilitic bony infections (osteitis, osteochondritis, and periostitis), CNS infections, and infection of virtually all other organ systems may be seen (Samuel et al., 2007). Although less than 1% of international adoptees are affected (Quarles and Brodie, 1998), screening is recommended for all (Committee on Infectious Diseases, American Academy of Pediatrics, 2009).

### Primary skin infections and their sequelae

Common skin conditions are also seen among children adopted internationally. These include scabies, pediculosis, molluscum contagiosum, and bacterial and fungal infections, usually presenting in a typical fashion (Committee on Infectious Diseases, American Academy of Pediatrics, et al., 2009, Miller et al., 2008, Quarles and Brodie, 1998, Van Schaik et al., 2009). Atypical presentation or treatment failure should raise suspicion of underlying immunosuppression, misdiagnosis, or infection with a resistant or endemic agent.

### Scabies

*Sarcoptes scabiei* infestation is extremely common in children living in tropical zones (Tong et al., 2011) and crowded orphanages. Some suggest that the majority of international adoptees with a history of institutional living are infected (Good et al., 2011). Adoptive parents often acquire medications and presumptively or empirically treat children in-country. Scabies in children adopted internationally usually has a classic presentation and responds to treatment with standard agents such as topical permethrin. However, improper or incomplete treatment may alter clinical appearance (Committee on Infectious Diseases, American Academy of Pediatrics, 2009). Secondary bacterial infection is common in the developing world, and in these cases, cultures are essential to choosing appropriate antibiotic treatment (Zhu et al., 2010) (see section on SSTIs). Persistent papules or pustules following treatment for scabies should trigger suspicion for infantile acropustulosis (Good et al., 2011) (see below).

### Infantile acropustulosis

Infantile acropustulosis is common in children adopted from overseas orphanages, and is thought to usually be a sequel of scabies infestation. Classically, children present within the first few months of life with intensely pruritic, vesiculopustular eruptions of the hands and feet. There may be history of a treated scabies infection. Acral surfaces are always affected, and lesions may also be seen on the dorsal hands and feet, limbs, trunk, and face. Eruptions last a week or more and recur at 1–3 month intervals. Episodes gradually decrease in frequency and severity, often disappearing by age 2 or 3 (Mazereeuw-Hautier, 2004, Tong et al., 2011).

Scabies infection is often difficult to distinguish from infantile acropustulosis. Key features which suggest infantile acropustulosis are early age of onset, recurrent nature, acral involvement, and absence of typical scabetic features, such as nodules and burrows. Laboratory findings are non-diagnostic, although hypereosinophilia and increased levels of serum IgE have been observed in some cases (Good et al., 2011, Tong et al., 2011).

Treatment is symptomatic, with topical corticosteroids considered the first line of treatment. Antimicrobial cleansers can prevent secondary infection, and antihistamines may alleviate pruritus. In severe or debilitating cases, dapsone may shorten the duration and progression of eruptions (Good et al., 2011).

### Skin and soft tissue infection (SSTI) and Staphylococcus aureus

SSTI such as impetigo and pyoderma are extremely common. Risk factors include scabies infestation, overcrowding, inadequate hygiene, and hot, humid weather— exposures common to many international adoptees in their home countries. Of these factors, scabies infestation has been shown to be particularly important (Zhu et al., 2010).

*Staphylococcus aureus* and *Streptococcus pyogenes* are the most frequent etiologic agents. Notably, infection and colonization with methicillin-resistant *S. aureus* (MRSA) among international adoptees is also well documented (Elstrøm et al., 2008, Gustafsson et al., 2007, Radtke et al., 2005). One Norwegian study, which performed routine MRSA screening upon hospital admission found colonization rates to be 70 times higher among international adoptees than children not adopted from abroad (Radtke et al., 2005). A Swedish study also demonstrated high rates of MRSA among international adoptees, and found that all infected children had a history of hospitalization prior to adoption (Elstrøm et al., 2008). Given that there has been an increase in the adoption of children with known medical needs, a history of hospitalization is an important factor to consider when deciding to screen for MRSA. Recent data suggests a rising prevalence of community acquired MRSA in less-developed countries, which may further contribute to high MRSA rates among adopted children (Zhu et al., 2010).

There are concerns that MRSA-colonized children may transmit infection to adoptive family members (Elstrøm et al., 2008, Mazereeuw-Hautier, 2004). A series of three Chinese children demonstrated MRSA transmission from one child to both parents and from a second child to her adoptive mother and infant sibling (Mazereeuw-Hautier, 2004). In a subsequent Swedish series, MRSA was transmitted from an infected child in only 3 of 13 adoptive families, and occurred when children were colonized with MRSA of the throat, anterior nares or peritoneum. Skin infection alone does not appear to carry risk of MRSA transmission to family members. Throat culture may be the most important screening test, as most MRSA positive adopted children were colonized in the throat (Elstrøm et al, 2008).

### Tinea

Tinea capitis is the most common dermatophyte infection in children under the age of twelve (Patel and Schwartz, 2011). Risk factors are similar to those for scabies and bacterial SSTIs. Tinea strains vary between countries. Therefore, culture is recommended prior to treatment. In one case report, two children adopted into an American family from Liberia were found to be infected with *Trichophyton soudanese*, which is rare in the US, but a common cause of tinea capitis in Africa (Markey et al., 2003).

## Conclusion

Health care providers who care for children adopted internationally should be aware of the spectrum of illnesses may be seen in this population, and should be prepared to encounter unusual situations. An appreciation for the unique exposure history and vulnerabilities of this population is critical to appropriate diagnosis and treatment.

## Figures and Tables

**Table 1:** Reported Conditions by Country/Region.

Conditions by country	Anomalies	Parasites	Rickets	+ PPD	SSTI	Viral Exanthem	Scars	Syphilis	HIV
China N = 452 (Miller and Hendrie, 2000) (10-J)	Ear pits (2), giant nevus (1), giant hemangioma (1), cleft (1) (Miller and Hendrie, 2000)	Toxoplasmosis (1), scabies (1) (Miller and Hendrie, 2000) (4) (Murray et al., 2005) Acropustulosis (2) (Tong et al., 2011)	Anecdotal reports of rickets in nearly every child (Rickets in Chinese Children, 2003)	1 case Lupus vulgaris from BCG vaccine (Thakur and Verma, 2011) *TB is endemic	6 (1 more with thrush) (Miller and Hendrie, 2000)	Measles (10) (Centers for Disease Control and Prevention (CDC), 2002), (9) (Centers for Disease Control and Prevention (CDC), 2007), Mumps (1) (Miller and Hendrie, 2000)^7^	Amputated finger from “rat bite” (Miller and Hendrie, 2000)	1 (Miller and Hendrie, 2000)	0^7^
Ethiopia N = 50 (Miller et al., 2008)	Anal fissures (3), ear pits (1), crumpled pinnae(1) (Miller et al., 2008) Dermal hypermelanosis (anecdotal) (Dinkins and Aronson, nd)	(32) trichuris trichiura (6), Hymenolepsis nana (3), Ascaris lumbricoides (2), hookworm (1), shistosoma mansoni (1) Dientamoeba fragilis (1) (Miller et al., 2008) Acropustulosis (1) (Tong et al., 2011)	No Data	8 (Miller et al., 2008) (36 had BCG) (Miller et al., 2008) *TB is endemic	23 (Miller et al., 2008)	Molluscum contagiosum anecdotally reported (Dinkins and Aronson, nd)	Female circumcision (26) (Miller et al., 2008)		0 (reports of positive parents) (Miller et al., 2008)
Russia N = 93 (Miller et al., 2005) (in Russian orphanages) N = 56 (in Soviet Union/Eastern Europe (Albers et al., 1997)	Fetal Alcohol Syndrome 19 (10%) (Miller et al., 2005)	Scabies (5) (Murray et al., 2005) Intestinal (51) (Albers et al., 1997)	41 (21%) (Miller et al., 2005)	5 (Albers et al., 1997) *TB is endemic	No Data	No Data	No Data	1 (Murray et al., 2005) history of congenital syphilis listed on pre-adoptive reviews in 15–20% (Albers et al., 1997)	0 (Albers et al., 1997)
South Korea *no data on adoption, numbers based on 2–3 years of in country reports (0–9 yrs old) (Lee and Allen, 2008)	No Data	“rarely infected” (Miller, 2005)	No Data	#2; 322 (2 yrs of data only) (Lee and Allen, 2008) *TB is endemic	No Data	#1; Measles (43) Mumps (2,050) Varicella (11,107; 2yrs of data only) (Lee and Allen, 2008)	No Data	0	No Data
Guatemala N = 103 (Miller et al., 2005)	CALM (20), ear pits (7), hemangioma (1) (Miller et al., 2005) 19 (18%) “features suggestive of prenatal alcohol exposure” (most “smooth philtrum”) (Miller et al., 2005)	Head lice (3), scabies (3) (Miller et al., 2005) Acropustulosis (1) (Tong et al., 2011)	1 (Miller et al., 2005)	7 (7%) (Miller et al., 2005) (57 BCG) (Miller et al., 2005 *TB is endemic	Yeast dermatitis (1) (Miller et al., 2005)	Roseola (1) (Miller et al., 2005)	Child abuse 1 (Miller et al., 2005)	1 (Murray et al., 2005)	0 (1 had mother who was positive) (Miller et al., 2005)

Given the sparse data published on the actual incidence of dermatologic diseases in internationally adopted children to the US, the data were gathered from a few choice reviews and a literature search for other specific documented cases. Full reviews were not available for South Korea and Russia.

A review of documented medical conditions in Russian orphanages and cases from a broader geographic area of Eastern Europe were included.

South Korea is unique in that pre-adoption living conditions are more favorable. A review on the prevalence of certain conditions in South Korea was used to find the most common conditions seen in this country.

It is the opinion of the authors, based on experience and anecdotal reports, that the incidence of many of these diseases is much higher than the literature reports would suggest. Improved disease reporting would lead to a better understanding of the conditions affecting this unique population.
